# Intestinal eosinophils, homeostasis and response to bacterial intrusion

**DOI:** 10.1007/s00281-021-00856-x

**Published:** 2021-04-30

**Authors:** Alessandra Gurtner, Ignacio Gonzalez-Perez, Isabelle C. Arnold

**Affiliations:** Institute of Experimental Immunology, University of Zürich, Winterthurerstr. 190, 8057 Zurich, Switzerland

**Keywords:** Eosinophils, Intestinal homeostasis, Bacterial infections, IFN-gamma, Protective immunity, Extracellular DNA traps, Microbiota, IBDs

## Abstract

Eosinophils are traditionally considered as end-stage effector cells involved in the pathogenesis of Th2 immune-mediated disorders as well as in the protection against parasite infection. However, this restricted view has recently been challenged by a series of studies revealing the highly plastic nature of these cells and implication in various homeostatic processes. Large numbers of eosinophils reside in the lamina propria of the gastrointestinal tract, at the front line of host defence, where they contribute to maintain the intestinal epithelial barrier function in the face of inflammation-associated epithelial cell damage. Eosinophils confer active protection against bacterial pathogens capable of penetrating the mucosal barrier through the release of cytotoxic compounds and the generation of extracellular DNA traps. Eosinophils also integrate tissue-specific cytokine signals such as IFN-γ, which synergise with bacterial recognition pathways to enforce different context-dependent functional responses, thereby ensuring a rapid adaptation to the ever-changing intestinal environment. The ability of eosinophils to regulate local immune responses and respond to microbial stimuli further supports the pivotal role of these cells in the maintenance of tissue homeostasis at the intestinal interface.

## Introduction

Eosinophils arise in the bone marrow from GATA-1-binding factor 1 (GATA-1) positive granulocyte-monocyte progenitors and develop to maturity in response to the cytokines GM-CSF, IL-3 and IL-5. Eosinophils are then released into the peripheral blood as terminally differentiated cells and rapidly migrate to their target tissues. While their half-life in the circulation is relatively short (estimated between 3 and 24 h) [1, 2], the survival of eosinophils upon migration into peripheral tissues is largely expanded, with turnover rates depending on distinct expression of the common γ-chain receptor in their target tissues [3]. Eosinophil chemotaxis is mainly driven by the binding of the chemokine eotaxin-1 (CCL11) to its CCR3 receptor, with CCL11 produced primarily by cells of stromal origin such as fibroblasts, smooth muscle cells and endothelial cells, but also by epithelial cells [4].

Small numbers of tissue resident eosinophils are found in multiple tissues under steady-state conditions, including the thymus, lung, uterus, mammary gland, adipose tissue and gastrointestinal (GI) tract. Interestingly, eosinophil numbers in the GI tract are substantially higher than in other tissues, accounting for 20–30% of the total intestinal leukocytes [5]. In addition to the pool of eosinophils present under steady-state conditions, eosinophils can further be recruited to sites of tissue damage in response to injury or exposure to allergen or pathogens, where they exert potent inflammatory effects through the release of cytokines, lipid mediators and cytotoxic granule proteins in a process known as “degranulation”. Eosinophil granules typically comprise eosinophil peroxidase (EPX), eosinophil cationic protein (ECP), major basic protein (MBP) and eosinophil-derived neurotoxin (EDN). The tight regulation of eosinophil effector functions is crucial for the development of beneficial immune responses and the simultaneous avoidance of excessive tissue damage. The abnormal presence of eosinophils in peripheral organs is therefore generally associated with disease. Eosinophilia is a major hallmark of allergic asthma in the airways [6, 7] and of allergic skin manifestations such as atopic dermatitis [8], but is also typical of chronic inflammatory conditions of the GI tract such as inflammatory bowel diseases (IBD) and eosinophil-associated gastrointestinal disorders (EGIDs—a family of conditions characterised by inappropriate GI eosinophil accumulation in the context of Th-2-driven immune polarisation) [9–11]. The tissue-damaging consequences of eosinophilia at these sites have been attributed to several factors released by activated eosinophils as they degranulate.

In the GI tract, eosinophils are scattered throughout the lamina propria of the stomach, small intestine, cecum and colon but are absent from the oesophagus. Eosinophils are recruited to the GI tract already during fetal life, before the establishment of the intestinal microflora [2]. The strategic location of intestinal eosinophils close to the mucosal interface and their expression of various Toll-like receptors (TLRs) at the cell surface suggests that they can sense and respond to microbial stimulation. Recent evidences show that eosinophils not only contribute to shape the composition of the intestinal microbiota—directly or indirectly via the modulation of mucosal immune responses—but also respond to bacterial components or bacterially derived metabolites. In this review, we concisely summarise the current understanding of eosinophil contribution to intestinal homeostasis and highlight recent literature investigating their ability to recognise and eliminate bacterial pathogens in the GI tract. We further discuss studies exploring interactions between eosinophils and the intestinal microbiota. As eosinophil contribution to the pathogenesis of Th2-oriented conditions such as asthma, allergy or infection with parasites has already been reviewed extensively [12–14], we focus here on evidence of their priming by the cytokine IFN-γ and potential implication in Th1 polarised immune settings such as following bacterial infections and bacterially driven inflammation.

## Eosinophil immunoregulatory function in intestinal homeostasis

The intestinal lamina propria is home to a dense and highly specialised mucosal immune system, comprising multiple T, B, innate lymphoid and myeloid cell subsets that act in concert with epithelial and stromal cell populations to mount effector immune responses against pathogens while avoiding deleterious responses to commensals. This dynamic crosstalk ensures the coexistence of the immune system with the microbiota in a mutually beneficial relationship, also known as “homeostasis”.

**Fig. 1:**
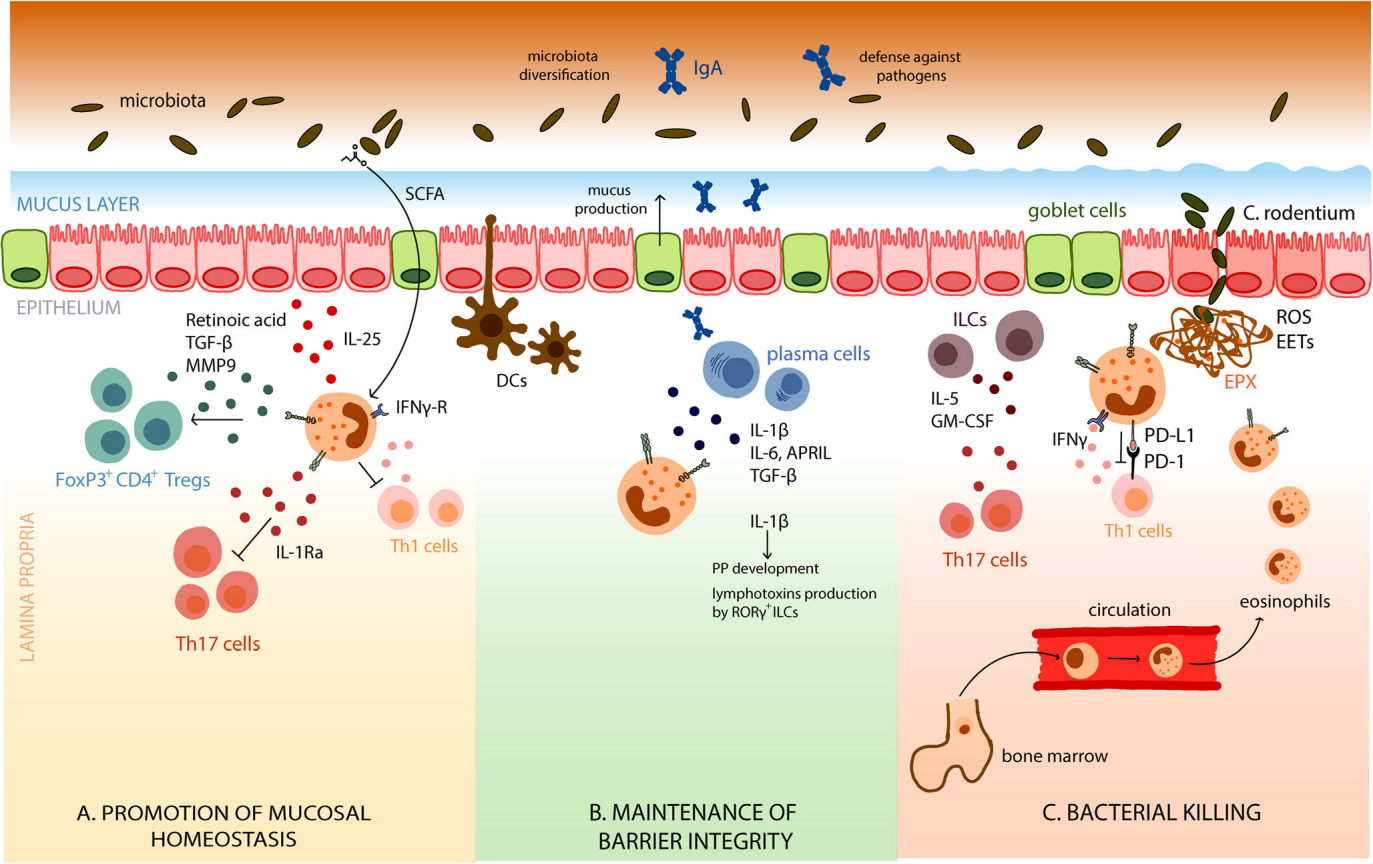
Roles of eosinophils in intestinal homeostasis and host protective immunity. The gastrointestinal tract is home to a large population of resident eosinophils. **a** Under physiological conditions, eosinophils integrate tissue-derived signals and bacterial metabolites to promote mucosal homeostasis. They restrict inappropriate Th1 responses in response to microbiota-derived signals and inhibit Th17 cell by secreting IL-1Rα. Eosinophils also promote the differentiation of regulatory T cells via the release of TGF-β, MMP9 and retinoic acid. **b** Eosinophils maintain epithelial barrier integrity by enhancing intestinal mucus secretion and supporting IgA-producing plasma B cells through the production of cytokines such as IL-1β, TGF-β and IL-6, leading to the diversification of the microbiota. Eosinophil-derived IL-1β also promote the development of Peyer’s patches and lymphotoxins production by RORγ^+^ ILCs. **c** In response to bacterial pathogens such as *C. rodentium* breaching the epithelial barrier, eosinophils are further recruited from the bone marrow to sites of tissue damage, where they are conditioned by IFN-γ to facilitate bacterial killing through the release of extracellular DNA traps (EETs) and associated cytotoxic granule proteins. Concomitantly, eosinophils also downmodulate Th1 responses via the expression of PD-L1

Eosinophils contribute to the maintenance of intestinal homeostasis in several ways. They preserve the epithelial barrier integrity by enhancing intestinal mucus secretion and eosinophil-deficient mice display decreased mucus-expressing goblet cells in the small intestine [15]. Eosinophils also support the maintenance of IgA-producing plasma B cells, which in turn promote the development of Peyer’s patches and modulate the composition of the intestinal microbiota [15, 16]. In addition, there is growing evidence on the role of eosinophils in regulating local immune responses, especially of T cells. Indeed, eosinophils down-regulated Th17 cells in the small intestine by secreting IL-1 receptor antagonist (IL-1Rα), a natural inhibitor of IL-1β [16]. Similarly, the frequencies of mucosal Th1, but not Th2 cells, were strongly increased in the GI tract of mice depleted of eosinophils in a microbiota-dependent manner [17] (Fig. 1). GI eosinophils were also reported to suppress Th2 responses in Peyer’s patches during intestinal infection by nematodes [18]. In contrast, enteric eosinophils promoted the initiation of Th2 immunity by controlling the activation and migration of CD103^+^ dendritic cells to draining lymph nodes in response to local EPX release in a model of food allergy [19]. These studies indicate that eosinophils might restrict inappropriate T cell responses to promote mucosal homeostasis, but that their function might be highly context dependent.

Eosinophil can also influence T cell responses indirectly, by promoting the differentiation of regulatory T cells (Tregs). Indeed, the analysis of eosinophil-deficient mice revealed a notable reduction in the frequencies of intestinal Foxp3^+^ Tregs correlating with decreased TGF-β activating factors MMP3 and MMP9 [20]. Similarly, intestinal eosinophils but not peripheral blood eosinophils induced the differentiation of naïve T cells into Foxp3^+^ Tregs cells in vitro through the release of TGF-β1 and retinoic acid [21]. Recent reports have described the existence of a unique microbiota-induced Treg subset expressing the nuclear hormone receptor RORγt, which controls intestinal inflammation [22–24]. Whether intestinal eosinophils also contribute to the differentiation of this specialised subset associated with enhanced suppressive functions still remains to be determined the effects of eosinophils on the maintenance of intestinal homeostasis.

## Pathways involved in eosinophil activation during bacterial infections

### Bacterial recognition pathways driving eosinophil activation

The mucosal immune system recognises microbial components and metabolites through several families of innate immune receptors, resulting in the production of cytokines, antimicrobial proteins and immunoglobulins (IgA) that maintain intestinal barrier integrity. Eosinophils are well equipped to sense and respond to bacterial stimulation. They express a vast varry of pattern-recognition receptors (PRRs) capable of recognising specific evolutionarily conserved microbial components called pathogen-associated molecular patterns (PAMPs), as well as damage-associated molecular patterns (DAMPs). PRR engagement on eosinophils activates intracellular signalling cascades leading to a broad range of responses, including the release of pro-inflammatory cytokines, chemokines, cytotoxic granule proteins, leukotrienes and reactive oxygen species, the upregulation of adhesion molecules increasing cellular trafficking as well as enhanced survival. Eosinophils express several families of PRR, including toll-like receptors (TLRs), RIG-I-like receptors (RLRs), nucleotide-binding oligomerisation domain-like (NOD-like) receptors as well as the receptor for advanced glycation end products (RAGE) [25]. An overview of the pattern-recognition receptors expressed on eosinophils are reviewed by Kvarnhammar et al. [25].

TLRs recognise PAMPs in different cellular compartments. TLR1, -2, -4, -5, -6 and -10 are positioned on the cell surface and primarily detect bacterial proteins, lipoproteins and polysaccharides. In contrast, TLR3, -7, -8 and -9 are located in endosomes, where they detect mostly viral nucleic acids. All TLRs except TLR8 have been detected in eosinophils at the mRNA or protein level. Peripheral blood eosinophils were reported to prominently express TLR7 [25], which recognises single-stranded RNA. While TLR7 signalling in eosinophils might contribute to host protection against viral pathogens, it remains to be determined whether it might also participate in the recognition of the viral component of the gut microbiome. The stimulation of human eosinophils with TLR2, TLR5 and TLR7 agonists led to the upregulation of intercellular adhesion molecule-1 (ICAM1) and of surface CD18 expression, together with the release of IL1β, IL-6, IL-8, CXCL1 and superoxides. These effects were mediated by the combined action of ERK kinase, PI3K kinase and NF-κB pathways [26]. In contrast, only the TLR2 agonist peptidoglycan (PGN) could induce eosinophil degranulation and ECP release [26]. In a study of Driss et al., both the live form of *M. bovis* bacillus Calmette-Guérin (BCG) and purified lipomannan promoted the synthesis of reactive oxygen species, EPX, ECP, TNF-α and α-defensins in a TLR2/Myd88-dependent manner [27]. Eosinophils further responded to bacterial lipopolysaccharide (LPS) stimulation, a TLR4 agonist, by enhanced survival and secretion of the cytokines GM-CSF, TNF-α and IL-8 [28] as well as by the release of ECP in a CD14-dependent manner [29]. However, the function of PRRs has mostly been studied in vitro, in isolated single cell systems, which neither take into account the tissue-specific characteristics of eosinophils nor recapitulate the local cytokine network. Indeed, it is likely that eosinophil response to selected bacterial triggers synergises with activating cytokine signals in a given tissue to increase TLR expression and signal transduction.

### Eosinophil priming through the Th1 cytokine IFN-γ

Among the cytokines priming eosinophil activation within the GI tract, IFN-γ might play an important role. IFN-γ is a pleiotropic cytokine produced predominantly by innate lymphoid cells (ILCs), natural killer (NK) cells, T-helper 1 (Th1) CD4^+^ T cells and cytotoxic CD8^+^ T cells. IFN-γ signalling through the IFN-γ receptor (IFN-γR) activates the Janus kinase (JAK)-signal transducer and activator of transcription 1 (STAT1) pathway to induce the expression of classical interferon-stimulated genes that have key immune effector functions, such as host defence against bacterial pathogens, modulation of immune and inflammatory responses as well as tumour immunosurveillance [30].

During microbial infection or tissue damage, an early burst of IFN-γ is produced by innate-like cells in response to the cytokines IL-12, IL-18 or following the activation of PRRs. This is followed by high and prolonged levels of IFN-γ produced by Th1 or CD8^+^ T cells upon TCR engagement in response to microbial peptide recognition [30]. IFN-γ is well known to regulate the function of multiple immune and non-immune cell types, including helper (Th) and follicular helper (Tfh) T cells, regulatory T (Treg) cells, B cells, innate-like lymphocytes, endothelial cells, stromal cells, adipocytes and neural cells [30]. IFN-γ is also a potent activator of different myeloid cells. In macrophages, IFN-γ mediates the polarisation to an ‘M1-like’ state [31], which results in their hyper-responsiveness to inflammatory stimuli and enhances their pro-inflammatory activity while promoting resistance to tolerogenic or anti-inflammatory factors [30]. IFN-γ further induces the local differentiation of monocytes into dendritic cells and macrophages at sites of infection [32]. In neutrophils, priming by IFN-γ increases oxidative metabolism, surface receptor expression and degranulation and strongly enhances their ability to kill pathogens [33].

By contrast, the effect of IFN-γ on eosinophil function is less well understood. IFN-γ was reported to promote the degranulation of human eosinophils and to enhance their production of superoxide anions following GM-CSF or IL-5 priming [34]. Further studies highlighted the role of IFN-γ in eosinophil primary granule mobilisation and piecemeal degranulation, a vesicle-dependent process allowing the selective release of part of their granule-stored contents. Indeed, IFN-γ induced the mobilisation of CD63 (a component of the late endosomal and lysosomal membranes also present in “secretory lysosomes,” [35]) to eosinophil peripheral membranes, together with the selective release of the chemokine CCL5 [36, 37]. The release of extracellular cell-free granules that retain their content of preformed cytokines and cationic proteins is typically observed during cytolysis, a nonapoptotic form of cell death [38] and is well documented in several human pathologies [39–41]. Interestingly, the extracellular granules of human, but not of mouse eosinophils, express the IFN-γ receptor α-chain (IFNGR1) on their membrane and remain ligand responsive, with the ability to differentially secrete their cationic proteins or cytokine contents [42, 43]. Thus, binding of IFN-γ to its receptor might continue to induce the local release of eosinophil granule proteins even in the absence of live eosinophils, thereby ensuring a long-lasting effect. Besides its effect on granule mobilisation, IFN-γ also supports eosinophil antimicrobial functions. The stimulation of mouse eosinophils with IFN-γ led to the killing of the parasite *L. amazonensis* in vitro in a reactive oxygen-dependent manner, but independent of their degranulation [44]. In addition, the priming of human eosinophils with IL-5 or IFN-γ is required for the release of eosinophil DNA traps in response to LPS stimulation [45].

**Fig. 2:**
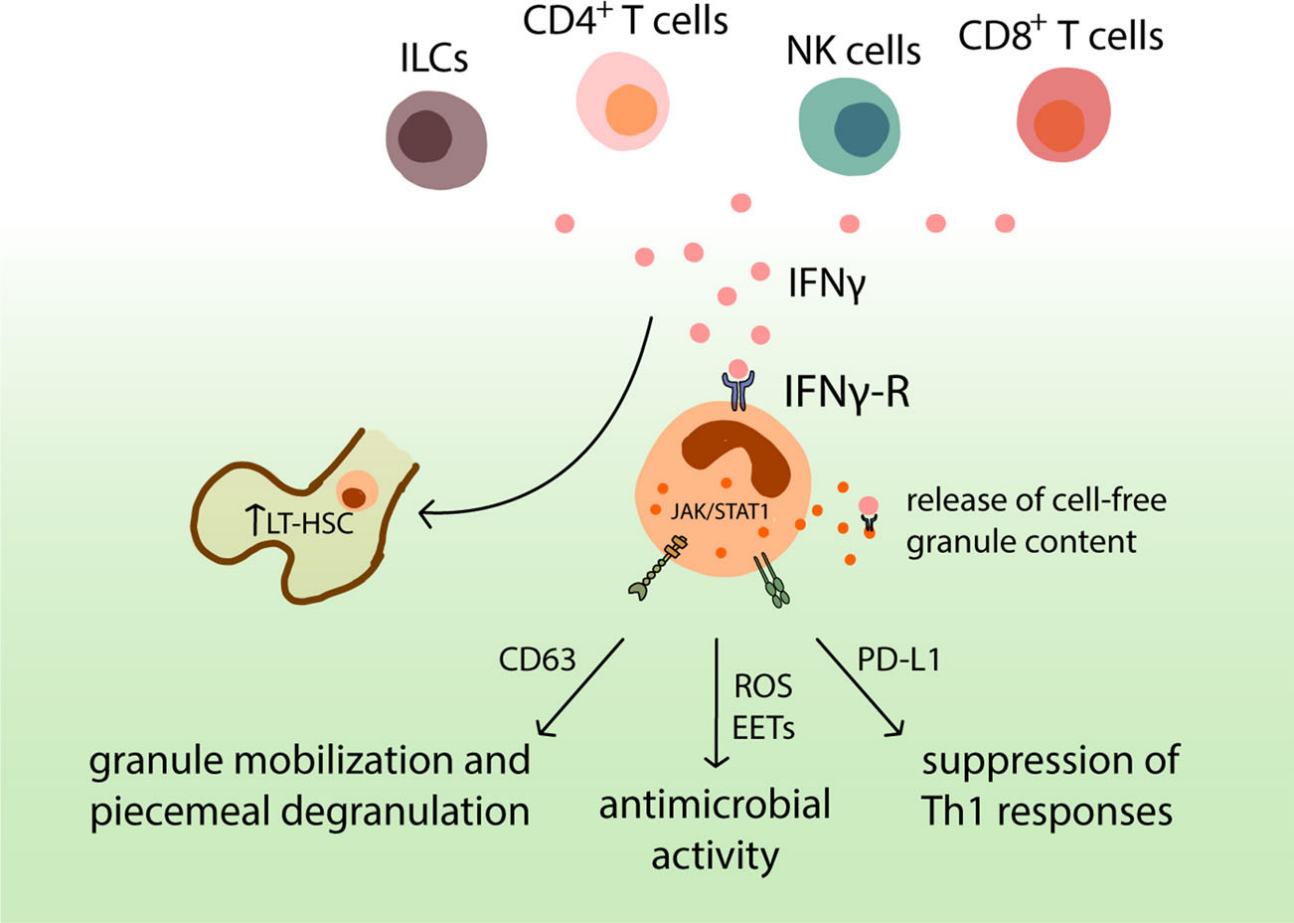
Eosinophil regulation by IFN-γ. Under homeostatic conditions, basal levels of IFN-γ are produced by innate lymphoid cells (ILCs), natural killer (NK) cells, T-helper 1 (Th1) CD4^+^ T cells and cytotoxic CD8^+^ T cells. IFN-γ induces mobilisation of CD63 to eosinophil peripheral membranes, followed by the piecemeal release of chemokines and granule proteins. Upon microbial infection or tissue damage, high and sustained expression of IFN-γ induces ROS-dependent antimicrobial activities and the release of EETs. During both acute and chronic bacterial infection, eosinophils prevent excessive inflammation by regulating local Th1 responses through the upregulation of PD-L1 in response to IFN-γ signalling. Besides its direct action on eosinophils, IFN-γ further promotes bone marrow eosinopoiesis by supporting the proliferation of long-term hematopoietic stem cell progenitors (LT-HSC). IFN-γ might also bind to the IFN-γR expressed at the surface of intact extracellular eosinophil granules, leading to the release of granular content even in the absence of live eosinophils

In the GI tract, basal levels of IFN-γ are expressed in response to specific commensal bacteria [46] and strongly increases during infection or inflammation. Our group has reported a key role for IFN-γ in the regulation of eosinophils in settings of acute and chronic bacterial infection [17]. We found that eosinophils are cell-intrinsically conditioned by IFN-γ levels in their residential tissues to promote homeostasis and restrict immunopathology by locally suppressing Th1 responses in an experimental model of *H. pylori* infection. Mice lacking the IFN-γR specifically in the eosinophil lineage mirrored the phenotype of eosinophil-deficient PHIL mice and exhibited higher frequencies of mucosal Th1 cells. The regulatory capacity of eosinophils toward Th1 cells depended at least in part on their PD-L1 expression but was independent of degranulation. Eosinophils isolated from infected tissues further exhibited a strong IFN-γ-associated genes signature characterised by the expression of *Pdl1*, *Cxcl10*, *Stat1* and *Ccl5.* The upregulation of these transcripts could be recapitulated in vitro upon stimulation of IFN-γ-primed eosinophils with live *H. pylori* and depended on the synergistic effect of both, *H. pylori* and IFN-γ stimulation, as neither signal alone was sufficient to drive differential gene expression [17]. Similarly, the transcriptional and proteomic analysis of colonic intra-tumoral eosinophils in a model of colitis-induced cancer revealed a strong IFN-γ-linked signature, indicating a key role for IFN-γ in activating eosinophils in experimental colorectal cancer [47]. It is interesting to note that in settings of chronic intestinal inflammation, IFN-γ producing T cells accumulate not only in the colon of mice, but also in the bone marrow [48]. There, IFN-γ increases the proliferation of long-term hematopoietic stem cell progenitors (LT-HSC) resulting in the enhanced production of downstream granulocyte-monocyte progenitors (GMPs), which also give rise to eosinophils [48, 49]. While this feed-forward mechanism may contribute to increase the eosinophil output observed during intestinal inflammation [50], it would be interesting to assess whether eosinophils are to a certain extent pre-activated by IFN-γ before reaching their target organs and whether this conditioning might impact their functional properties. The potential effects of IFN-γ signalling on eosinophil functions are summarised in Fig. 2.

## Eosinophils in host protective immunity against bacterial pathogens

Eosinophils have traditionally been associated with protection against parasitic helminth infections. However, emerging evidences show that eosinophils are also involved in the recognition and elimination of other pathogens such as viruses, fungi and bacteria. Eosinophil bactericidal activity might be particularly important at mucosal interfaces and in environments with high bacterial stimulation such as in the GI tract. In situations where the epithelial barrier integrity is compromised—such as during infection or chronic inflammation—the release of cytotoxic granules in association with EETs might provide a second physical barrier aimed at limiting bacterial invasion, but might also contribute to the pathogenesis of chronic inflammatory conditions such as IBD [45].

### Eosinophils in inflammatory bowel diseases (IBDs)

IBDs are chronic relapsing disorders affecting the gastrointestinal tract and associated with high morbidity. The two main forms of IBDs, Crohn’s disease and ulcerative colitis, are characterised by intestinal inflammation and epithelial injury. The precise aetiology of IBDs is still unclear, but chronic inflammation seems to arise from an abnormal immune response against the microorganisms of the intestinal flora in genetically susceptible individuals, resulting in the breakdown of intestinal homeostasis. IBD patients therefore often present signs of microbial imbalance, intestinal barrier dysfunction and dysregulation of the intestinal mucosal immune system. As opposed to primary EGIDs, which feature a predominant eosinophilic infiltrate, IBDs are characterised by a heterogeneous infiltration of inflammatory leukocytes, together with a marked increase in eosinophil numbers resulting from enhanced production of eotaxin 1 in the lamina propria by colonocytes, macrophages or B cells [51]. Strong evidence suggests that eosinophils play a cardinal role in the pathogenesis of IBDs by promoting tissue damage through excessive degranulation, likely in an attempt to protect the host from pathogen incursion [52]. Intestinal eosinophil densities or faecal granule protein levels directly correlate with disease severity [53], while eosinophil degranulation at sites of active mucosal inflammation is commonly reported [54, 55]. Interestingly, eosinophils seem to a certain extent pre-activated in the circulation of IBD patients, suggesting that mediators such as inflammatory cytokines and chemoattractants might prime their activation systemically [56]. Despite circumstantial evidence for a pathogenic role of eosinophils in IBD, the observation that high levels of activated eosinophils persisted in the lamina propria of UC patients in disease remission [57] suggests that tissue eosinophilia cannot be solely linked to eosinophil pro-inflammatory activities and might be highly context-dependent. The difficulty of attributing a definitive functional contribution of eosinophils to the pathogenesis of IBD is further illustrated in experimental models of intestinal inflammation, where eosinophil deficiency resulted in either ameliorated or worsened inflammation depending on the model used [50, 58, 59].

### Antibacterial properties of eosinophil granule proteins

The importance of eosinophils in host protection against bacterial pathogens is illustrated by Linch et al. describing the protective effect of eosinophilia in IL-5 transgenically engineered mice against *P. aeruginosa* [60]. While eosinophil-deficient mice were highly susceptible to peritonitis following *P. aeruginosa* infection and exhibited impaired bacterial clearance, the presence of eosinophilia or the adoptive transfer of eosinophil granule extracts reduced the bacterial load in vivo [60]. The antibacterial properties of eosinophil granule proteins are well documented for ECP, MBP, and EPX and are reviewed in Gigon et al. [61].

ECP is uniquely expressed in eosinophils and exerts potent antibacterial properties against both Gram-negative and Gram-positive strains independently of its ribonuclease activity [62]. Elevated levels of serum ECP are often found in patients with bacterial infections, leading to the conclusion that eosinophil activation in this setting results in the preferential mobilisation of ECP [63, 64]. The bactericidal effects of ECP can be related to its membrane disruption capacity [65–67] and ability to bind the bacteria-wall components LPS and PGN with high affinity, leading to membrane depolarisation [68]. ECP also induces bacterial aggregation and forms amyloid aggregates in vitro [68, 69], potentially enhancing pathogen agglutination and killing at the infection foci. Similarly, MBP-1 exhibits non-selective cytotoxicity toward bacteria by binding to and permeabilising bacterial membranes [67, 70]. Upon release, MBP-1 aggregates and forms amyloids that facilitate antimicrobial activity*.* Interestingly, large MBP amyloid plaques present in the tissues of eosinophilic patients are characterised by decreased cytotoxicity and were proposed to result from a feedback mechanism aimed at limiting tissue damage under pathological conditions, while providing a scaffold for the recruitment of innate immune cells [71]. Indeed, the stimulation of human eosinophils with MBP leads to further degranulation and release of IL-8 [72], a pro-inflammatory cytokine with potent chemoattractant properties for innate immune cells. EPX is a cationic haloperoxidase that shares 70% amino acid homology with the better characterised neutrophil myeloperoxidase [73]. EPX catalyzes the oxidation of halide and pseudohalides ions present in the plasma together with hydrogen peroxide to form highly cytotoxic hypohalous acids involved in bacterial killing [10]. EPX interacts with the superoxide generated by the NADPH oxidase to provide the bactericidal activity of eosinophils [74, 75].

### Eosinophil-derived extracellular DNA traps

The elimination of bacterial pathogens mainly occurs extracellularly through the release of eosinophil granule proteins at high local concentration, which are toxic to both pathogens and neighbouring cells. Interestingly, granule proteins can also be found attached to DNA released from activated eosinophils. These so-called extracellular traps (EETs) form a localised scaffold that captures both granule proteins and bacteria, thereby ensuring the targeted killing of pathogens while limiting cytotoxic damages to the surrounding tissues. While the deposition of extracellular DNA by eosinophils contributes to the innate host defence machinery, it also likely plays an important role in the development of certain pathologies, particularly in settings of chronic, unresolved inflammation. Evidence of eosinophil-derived extracellular DNA depositions are observed in multiple human pathologies, including Crohn’s disease [45], allergic asthma [76], atopic dermatitis [77] and eosinophilic esophagitis [78], as well as following bacterial infections [45, 79]

The release of EETs can be initiated by several mechanisms, including TLR-, cytokine-, chemokine- and adhesion receptor-mediated signal transduction pathways [80]. The mechanism of EET formation and origin of the released DNA are still a matter of debate and are discussed elsewhere [81]. Yousefi et al. first demonstrated the catapult like release of mitochondrial DNA in response to bacteria such as *E. coli*, which led to rapid bacterial killing in a phagocytosis-independent manner [45]. The release of EETs could further be recapitulated in vitro by stimulating human eosinophils with LPS, complement receptor 5a or eotaxin following IL-5 and/or IFN-γ priming. This process depended on reactive oxygen species and was independent of cell death or apoptosis [45]. Degranulation and EETs formation seem to rely on different molecular pathways, as the release of eosinophil granule proteins was reported to precede the release of DNA, implying that their association takes place in the extracellular space [82].

**Fig. 3:**
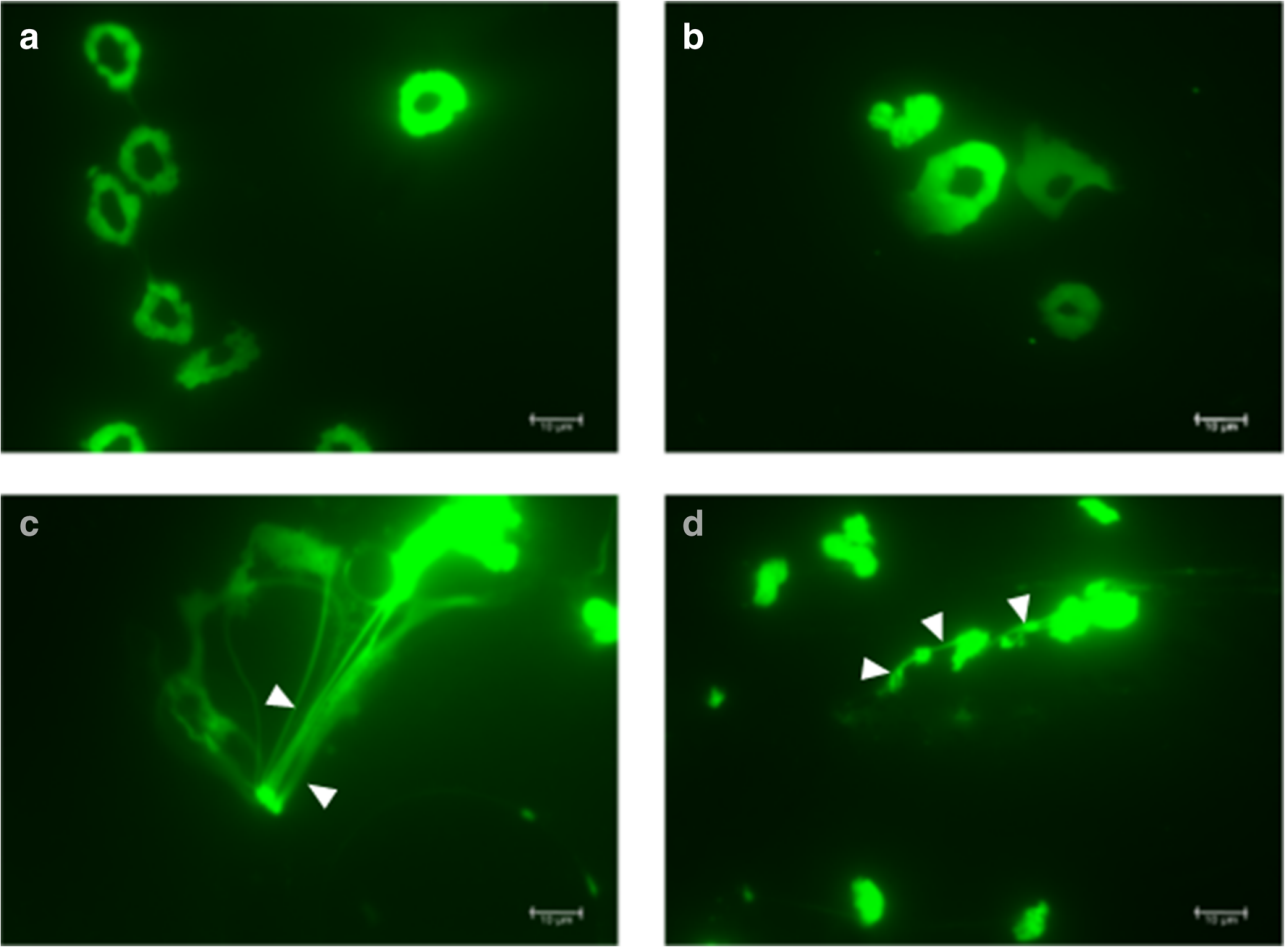
Selected bacterial species promote eosinophil DNA extracellular trap formation (EETs). **a**–**d** Eosinophils were isolated from the spleens of IL5-tg mice and sorted by flow cytometry. Eosinophils were then infected with live *Helicobacter hepaticus* (**b**), *Citrobacter rodentium* (**c**), *Salmonella typhimurium* MCI (**d**) for 15 min at 37 °C or were left untreated (**a**). Cells further received 5 μM/well of the nucleic acid stain Sytox green (Invitrogen) 5 minutes after infection, and were visualised for extracellular trap formation on a Leica DM6 B microscope. White arrows point toward EETs. The formation of EETs was clearly visible in *C. rodentium* infected eosinophils, while less marked DNA filaments and nuclear disintegration was observed following *S. typhimurium* infection. In contrast, EETs formation was not observed in response to *H. hepaticus* infection or in uninfected eosinophils. Scale bar represents 10 µm

The relevance of EETs in eosinophil bactericidal activities was further demonstrated in vivo. In a model of post-caecal ligation and puncture, IL-5 transgenic but not wild-type mice displayed substantial deposition of extracellular DNA indicative of EETs, which protected mice against microbial sepsis [45]. In a model of acute colitis induced by the pathogen *C. rodentium*, eosinophils were rapidly recruited to the colonic lamina propria upon bacterial challenge, where they degranulated and released EETs associated with EPX (Fig. 1) [17]. In the absence of eosinophils, mice displayed impaired bacterial clearance and increased susceptibility to the pathogen, as evidenced by stronger Th1/Th17 responses and immunopathology. *C. rodentium* was further highly susceptible to killing by activated eosinophils *in vitro* and bacterial viability coincided with the release of EPX and the formation of EETs [17]. Interestingly, the triggering of EETs leading to bacterial killing seems to be highly dependent on the nature of the bacterial stimuli, as only selected bacterial species—such as *C. rodentium* and *S. Typhimurium* but not *H. hepaticus* or *H. pylori*—are capable of provoking EETs in vitro (Fig. 3). In addition, the induction of lytic cell death and extracellular DNA release by *S. aureus* depended on the expression of its Hla virulence factor [83]. It is thus tempting to speculate that the inactivation of specific bacterial virulence factors, a strategy commonly employed by pathobionts such as *H. pylori* to escape immune recognition [84], might also contribute to avoid eosinophil-mediated killing. Future studies addressing the mechanisms through which selective bacterial species or virulence factors are recognised by eosinophils and lead to distinct functional responses would thus help to better understand the role of eosinophils in bacterial infections and bacterially driven pathologies.

## Eosinophils and the microbiota

The GI tract is home to the densest microbial communities known. Bacteria outnumber host cells by a factor of 10 and exist in a highly adapted and mutualistic relationship with the host, contributing to nutrition, immune system development and function, as well as protection from pathogens. Imbalance in the microbiota composition is referred to as “dysbiosis” and is associated with the pathogenesis of both intestinal and extra-intestinal disorders [85]. Dysbiosis is also commonly observed in IBD patients and is characterised by an overall reduced bacterial diversity together with an increased abundance of mucolytic, sulfate reducing and pathogenic bacteria, which contribute to alter mucosal integrity and promote inflammation [86].

Eosinophils are located in the GI lamina propria—separated from our microbial residents by just a single layer of epithelial cells. While several studies have reported an important role for eosinophils in maintaining a homeostatic composition of the intestinal microbiota, much less is known about how microbiota-derived factors and metabolites might regulate local eosinophil functions.

### Modulation of the intestinal microbiota by eosinophils

Immunoglobulin A (IgA) is the dominant antibody isotype found in mucosal secretions and promotes host-microbiota symbiosis by impacting the composition and density of intestinal bacterial communities. IgA is secreted into the gut lumen, where it binds to and ‘coats’ specific members of the microbiota. While the functional consequences of IgA binding are still incompletely understood, impaired IgA production is related to decreased overall microbial diversity and shifts in the relative abundances of specific bacterial taxa [87–89]. Chu et al. first reported that eosinophil-deficient mice generated through the deletion of a high-affinity GATA-binding site in the GATA-1 promoter (ΔdblGATA-1) [90] have impaired generation and maintenance of IgA plasma cells in the small intestine [20]. In this study, eosinophils directly regulated GI IgA production through their expression of IL-6, APRIL and TGF-β via TLR-mediated signalling, resulting in reduced secretory IgA levels, less IgA adherence to faecal bacteria and a reduction of Gram-positive bacteria, possibly Firmicutes [20]. Similarly, Jung et al. described the reduced expression of secretory IgA and bacterial imbalance associated with impaired Peyer’s patches development in the absence of eosinophils, but suggested an indirect regulatory mechanism implicating eosinophil-derived IL-1β and lymphotoxins [15]. A schematic depiction of the pathways through which eosinophils support the generation of IgA is represented in Fig. 1. In contrast to these reports, later studies found only modest or no differences in the numbers of IgA-secreting plasma cells in eosinophil deficient mice, proposing that factors such as the genetic background or age of mice might account for these differences [91, 92]. More recently, Beller et al. reported that mucosal IgA production was in fact determined independently of eosinophils by specific members of the intestinal microbiota themselves [93]. The cohousing of eosinophil-deficient mice with their wild-type counterparts prior to analysis led to the equalisation of their microbiota and normalisation of IgA levels, in contrast to non-cohoused animals. By comparing the microbiome of eosinophil-deficient and wild-type mice, bacteria enriched for the genus Anaeroplasma were further identified as major driver of TGF-β expression in intestinal T follicular helper cells leading to IgA class switching and enhancing mucosal IgA levels [93]. Interestingly, the microbial analysis of cohoused ΔdblGATA-1 and wild-type littermates revealed that the absence of eosinophils primarily affect the composition of mucus-resident bacterial species in the large and small intestine, with little change in mucosal IgA [92]. These observations implicate that eosinophils might also regulate the microbiota by mechanisms independent of IgA, especially the bacteria most hyperlocal to the gut barrier. The impact of eosinophils on the microbiota composition might be triggered directly through the secretion of anti-bacterial factors or indirectly, by promoting the production of epithelium-derived anti-bacterial peptides or through the regulation of local immune responses. While eosinophils clearly seem to participate in the dynamic modulation of intestinal bacterial communities, the mechanisms behind these activities are likely to extend beyond the action of IgA alone.

### Eosinophil response to microbial signals

Besides their impact on the microbiota composition, eosinophils also respond to microbial stimuli in several ways. While eosinophils can directly encounter specific pathogens capable of dwelling through the dense GI mucosal layer [17], direct contacts between eosinophils and commensal bacteria are less likely to occur under homeostatic conditions due to the strict compartmentalisation of the intestinal microbiota to the mucosal surface. Eosinophils might thus recognise bacterial metabolites or might be conditioned indirectly via microbiota-derived signals acting via the epithelium. Indeed, a cross-talk between eosinophils and intestinal epithelial cells has been proposed to limit *C. difficile* infection in an IL-25-dependent manner [94]. Buonomo et al. reported that mice treated with the microbiota-regulated cytokine IL-25 were protected from lethal *C. difficile* infection in an eosinophil-dependent manner. Mice lacking eosinophils suffered profound epithelial destruction unrelated to the levels of bacterial colonisation, IL-4, mucin or IgA [94]. The results suggest that in response to microbial signals, epithelial-derived IL-25 promotes eosinophil homeostatic function that maintains epithelial barrier integrity. In addition, a recent report indicated that eosinophil survival was negatively regulated by the bacterial metabolite butyrate [84]. Butyrate is a short chain fatty acid (SCFA) produced through the microbial fermentation of dietary fibres in the lower intestinal tract and has received much attention recently for its beneficial effects on intestinal homeostasis and anti-inflammatory properties [85]. Patients with IBDs have an altered gut microbial composition and a concurrent reduction in butyrate-producing bacteria [95]. Interestingly, both mouse and human eosinophils express strikingly high levels of the SCFA receptors GPR43 and GPR41 (free fatty acid receptors 2 and 3, respectively) in comparison to other leukocytes [96, 97]. In vitro, butyrate induced eosinophil apoptosis and attenuated their migratory and adhesion capacities. Butyrate further alleviated allergic airway inflammation by limiting eosinophil trafficking in vivo [85]. While the physiological relevance of these observations in the context of intestinal homeostasis still needs to be further explored, it suggests that eosinophil survival and possibly functional polarisation in the lamina propria might be directly controlled by commensal-derived metabolites.

The critical interplay between the microbiota and intestinal eosinophils in shaping homeostatic immune processes is further illustrated in studies using germ-free (GF) mice. In a recent reported, Jiménez-Saiz and co-workers report a significantly higher frequency of eosinophils in the intestines of GF than of specific pathogen free control animals [98]. The intestinal eosinophils of GF mice also exhibited a striking reduction of cytoplasmic granule size and content, further supporting a role for the microbiota in shaping the phenotype and density of local eosinophil populations. Interestingly, GF mice also displayed increased eosinophil frequencies at other mucosal sites such as the lung or the vaginal tract but not in sterile tissues such as spleen or uterus, which could be normalised by microbiota repletion. [98]

While microbiota-derived signals seem to modulate different aspects of eosinophil biology, the precise nature of these signals and whether they might synergise with local cytokine networks to enforce a niche-specific spectrum of activities resulting in distinct functional subsets still needs to be investigated further.

## Concluding remarks

Eosinophils are an integral part of the resident intestinal immune system, conferring protection against invading pathogens while exerting subtle regulatory effects on local immune cells. In addition to their homeostatic functions, eosinophils also promote inflammation and tissue damage through their excessive degranulation in settings of chronic, unresolved inflammation. Despite these evidences, eosinophils are often overlooked. With the development of new experimental models of eosinophil deficiency and tools specifically targeting the eosinophil lineage, the extent of their contribution to tissue homeostasis and protective immunity has slowly begun to be revealed. However, these experimental strategies may underestimate the phenotypic diversity of tissue-resident or disease-associated eosinophils. Further studies relying on modern technologies such as proteomics, single cell sequencing or high-dimensional flow-cytometry might thus help to elucidate the intricate molecular pathways defining eosinophil heterogeneity along the GI tract. The identification of distinct functional subsets might further lay the basis for exploiting new pharmacological strategies to manipulate eosinophil activities in pathologies such as IBD and provide mechanistic insights that may be applicable to other eosinophil-mediated disease contexts.
